# Validation of flavivirus infectious clones carrying fluorescent markers for antiviral drug screening and replication studies

**DOI:** 10.3389/fmicb.2023.1201640

**Published:** 2023-09-15

**Authors:** Liubov Cherkashchenko, Nathalie Gros, Alice Trausch, Aymeric Neyret, Mathilde Hénaut, Gregor Dubois, Matthieu Villeneuve, Christine Chable-Bessia, Sébastien Lyonnais, Andres Merits, Delphine Muriaux

**Affiliations:** ^1^CEMIPAI UAR3725 CNRS, University of Montpellier, Montpellier, France; ^2^Institute of Technology, University of Tartu, Tartu, Estonia; ^3^IRIM UMR9004 CNRS, University of Montpellier, Montpellier, France

**Keywords:** flaviviruses, infectious clone, Zika virus, dengue virus, Kunjin virus, fluorescent marker and reporter genes, reverse genetics

## Abstract

Flaviviruses have emerged as major arthropod-transmitted pathogens and represent an increasing public health problem worldwide. High-throughput screening can be facilitated using viruses that easily express detectable marker proteins. Therefore, developing molecular tools, such as reporter-carrying versions of flaviviruses, for studying viral replication and screening antiviral compounds represents a top priority. However, the engineering of flaviviruses carrying either fluorescent or luminescent reporters remains challenging due to the genetic instability caused by marker insertion; therefore, new approaches to overcome these limitations are needed. Here, we describe reverse genetic methods that include the design and validation of infectious clones of Zika, Kunjin, and Dengue viruses harboring different reporter genes for infection, rescue, imaging, and morphology using super-resolution microscopy. It was observed that different flavivirus constructs with identical designs displayed strikingly different genetic stabilities, and corresponding virions resembled wild-type virus particles in shape and size. A successful strategy was assessed to increase the stability of rescued reporter virus and permit antiviral drug screening based on quantitative automated fluorescence microscopy and replication studies.

## Introduction

The Flaviviridae family includes four genera: *Flavivirus*, *Pestivirus*, *Pegivirus*, and *Hepacivirus*.^1^ The genus *Flavivirus* includes several arthropod-borne viruses that usually infect insects but some can be responsible for a significant number of human diseases primarily caused by dengue virus (DENV), Zika virus (ZIKV), West Nile virus (WNV), yellow fever virus (YFV), Japanese encephalitis virus (JEV), and tick-borne encephalitis virus (TBEV). In the majority of the cases, transmission occurs horizontally between mosquitoes (often *Aedes* or *Culex*) or ticks and vertebrate hosts, whereas mammalians often serve as reservoirs and thus contribute to the adaptation of viruses (Weaver and Barrett, 2004; Pierson and Diamond, 2020). In this study, we focused on investigating three members of the genus *Flavivirus*: DENV (serotypes 2 and 4); ZIKV; and Kunjin virus (KUNV), an Australian subtype of WNV.

The flavivirus genome is a positive-strand RNA of approximately 11,000 bases containing a single open reading frame (ORF) that is flanked by two untranslated regions (UTRs) located at the 5′ and 3′ ends of the virus genome. The genome has a cap structure located at the 5′ end but lacks the 3′ poly(A) tail. The absence of poly(A) is compensated for by the interaction of the 3’UTR with poly(A)-binding protein (PABP). The *Flavivirus* ORF encodes for a polyprotein precursor of viral proteins that is cleaved by viral and host proteases into three structural proteins [C (capsid), prM(M) (membrane), and E (envelope)] and seven non-structural (NS) proteins (NS1, NS2A, NS2B, NS3, NS4A, NS4B, and NS5). The NS proteins play multiple roles in the infection cycle, including in viral RNA replication, the assembly of viral particles, and the evasion of the host immune response. The structural proteins are involved in the formation and release of viral particles (Mazeaud et al., 2018; Choi, 2021).

The spectrum of clinical manifestations of flavivirus infection ranges from mild (asymptomatic) illness to severe disease (Pan et al., 2022). Such a divergence is mostly caused by the different tropisms of viruses or/and their ability to counteract the host immune response. There are approximately 400 million flavivirus infections every year, a large majority of which are caused by four genotypes of DENV (DENV1-4), making it one of the most medically important causative agents of human diseases (Guabiraba and Ryffel, 2014). The clinical presentation of DENV infection includes the development of dengue fever and other more severe diseases—dengue hemorrhagic fever and dengue shock syndrome (Gould and Solomon, 2008; Khanam et al., 2022). By contrast to DENV, symptomatic infection by ZIKV is mostly associated with mild symptoms, such as headache, joint pain, or cutaneous rash. In some cases, ZIKV infection in women during pregnancy can be considered as one of the causative agents of microcephaly in newborns and has been associated with progression into Guillain–Barré syndrome (Wen et al., 2017; Barbi et al., 2018; Lima et al., 2019). Interestingly, WNV has a wider range of vertebrate hosts, and horses and humans represent dead-end hosts. WNV comprises at least seven genetic lineages. KUNV belongs to lineage 1b of WNV and is endemic to Australia. Infection with KUNV mostly leads to the development of mild symptoms in humans but the infection is lethal for horses (Colpitts et al., 2012). Currently, there are no specific treatments for flavivirus infections or efficient methods for controlling the spread of their arthropod vectors. Vaccines capable of preventing infection are available for YFV, JEV, and TBEV. The development of a vaccine for DENV is hampered due to the immunological cross-reaction between different serotypes; in such cases, virus may escape neutralization, which leads to an antibody-dependent enhancement (ADE) believed to be responsible for the establishment of severe forms of DENV disease (van Leur et al., 2021).

Antiviral drug discovery for flaviviruses requires the development of robust screening and antiviral activity assays, which have been the focus of numerous studies. To date, diverse methods have been successfully applied to quantify the effect of antivirals on flavivirus infection; however, they remain time-consuming and expensive (Che et al., 2009; Gurukumar et al., 2009; Shum et al., 2010; McCormick et al., 2012; Cruz et al., 2013; Faye et al., 2013; Wilson et al., 2017; Xu et al., 2017). Therefore, the development of new approaches using reverse genetics of flaviviruses, such as the generation of infectious clones and recombinant flaviviruses harboring marker genes (encoding for fluorescent or luminescent reporters), is necessary. As a part of the study, analysis of the properties of DENV, ZIKV, and KUNV reporter viruses allowed the development of efficient bioluminescence or image-based high-throughput assays applicable to drug discovery (Zou et al., 2011; Schoggins et al., 2012; Fischl and Bartenschlager, 2013; Koishi et al., 2018; Li et al., 2020; Zhang et al., 2021). Nevertheless, genetic stability and durable reporter expression during long-term passage in tissue culture remained a key issue (Aubry et al., 2015).

In the current study, we present the data related to the approaches used for the construction of wt infectious clones of KUNV, DENV2, and DENV4 as well as versions harboring fluorescent and luminescent reporters based on a strategy previously applied to ZIKV (Mutso et al., 2017). Following an assessment of the growth kinetics of viruses rescued from these clones, the genetic stability of the marker-coding viruses was analyzed by monitoring the fluorescence or luminescence signals from the cells infected with the corresponding viruses. Herein, we characterized the structures of the virions and the variations in their sizes and shapes, as well as their distribution in cellular compartments, using transmission electron microscopy (TEM) on fixed infected cells and atomic force microscopy (AFM) on live viruses (Lyonnais et al., 2021). Finally, we validated the use of the obtained recombinant viruses in fluorescent or luminescent-based drug screening assays by testing NITD008 as a reference molecule harboring pan-flavivirus activity.

## Results

### Design and construction of icDNA clones of DENV2, DENV4, and KUNV

Rescue of the virus from the infectious cDNA (icDNA) clone of an RNA virus enables various modifications and/or manipulations of the RNA genome. The development of reverse genetic systems for different RNA viruses has advanced over the last decades. The RNA transcripts derived from cDNAs of positive-strand RNA viruses are considered to be infectious, i.e., their transfection into susceptible cells results in the replication and successful recovery of the infectious viruses (Ruggli and Rice, 1999). For most positive-strand RNA virus families this approach is relatively straightforward; however, for some viruses, the development and use of reverse genetics is more challenging. Flaviviruses represent an example of the latter. It has been shown that some sequences from flavivirus genome encode proteins with a high level of cytotoxicity for *Escherichia coli*, the bacteria most commonly used to propagate plasmids containing viral icDNAs (Zheng et al., 2016). The expression of such proteins via the activity of cryptic bacterial promoters found in the cDNAs of flaviviruses is therefore harmful to the bacteria harboring the corresponding plasmids, leading to counterselection resulting in the instability of icDNA plasmids.

Here, we aimed to construct icDNAs based on the NCBI sequences for DENV2 (GenBank: U87411.1), DENV4 (GenBank: AF326573.1), and KUNV (GenBank: AY274504) using a strategy described previously for ZIKV (GenBank database: KJ776791) (Aubry et al., 2015). Briefly, the SP6 promoter was placed upstream of the sequence corresponding to the 5′ end of the virus genome while a cleavage site of restriction endonuclease was placed immediately downstream of the sequence corresponding to the 3′ end of the genome. This allowed run-off *in vitro* transcription to be conducted to obtain transcripts corresponding to the viral genome RNA that could be used for the efficient rescue of infectious viruses. The sequences corresponding to virus genomes were assembled from synthetic DNA fragments; assembly was performed in a single-copy plasmid to ensure the efficient propagation of cloned cDNAs in bacterial cultures and to prevent possible re-arrangements in the cDNAs of flaviviruses (Pu et al., 2011). No instability issues were observed during plasmid construction and the propagation of plasmids containing cDNAs of KUNV, DENV2, and DENV4. Of note, this approach failed with the icDNA of DENV3, probably indicating extreme toxicity of the latter plasmid for *E. coli.*

**Figure 1:**
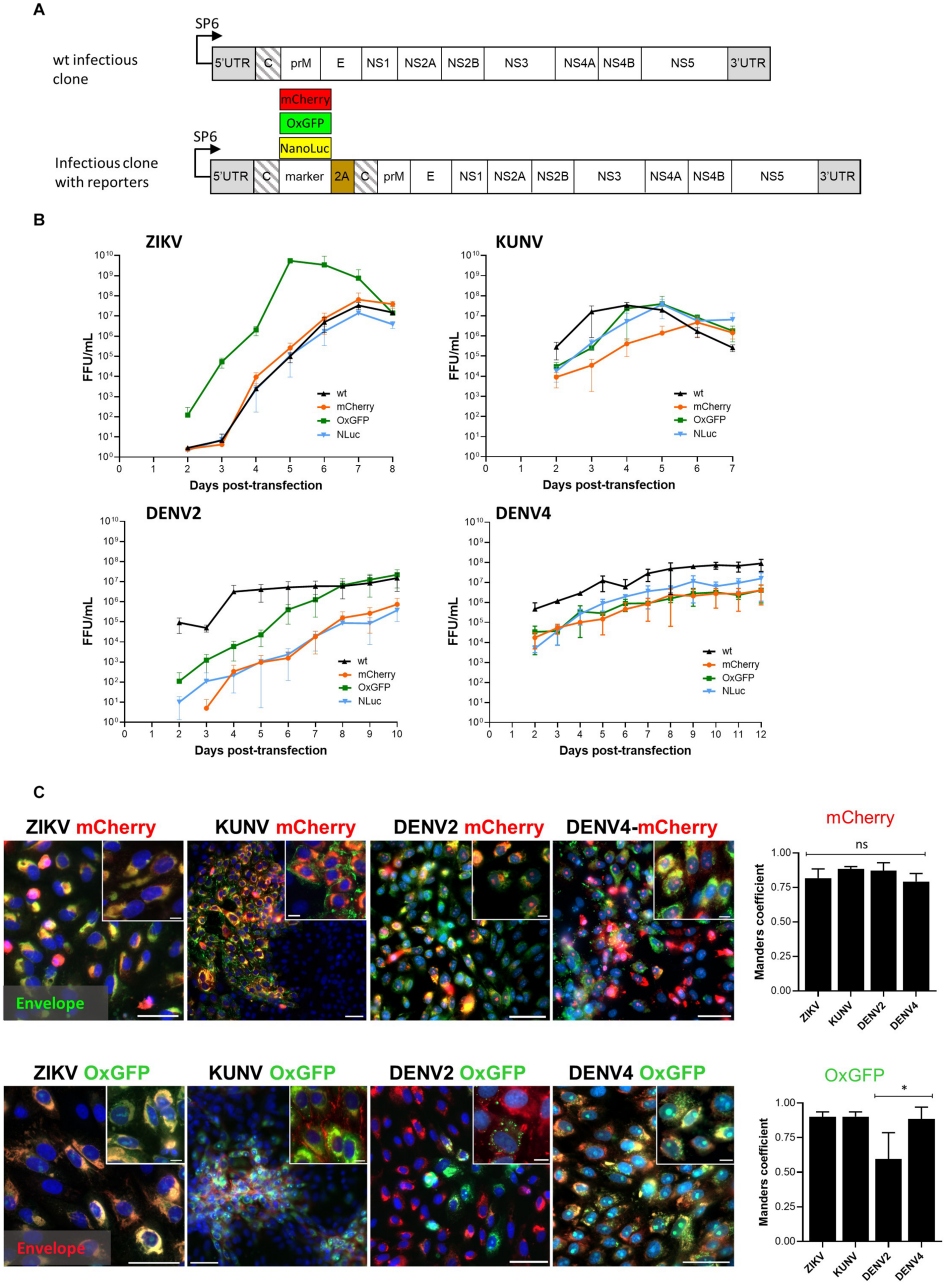
Design of the wt and reporter containing the icDNA of flaviviruses. **(A)** Schematic of the viral constructs used in the study. The reporter (mCherry, oxGFP, or NLuc) and foot-and-mouth-disease virus 2A sequence (2A) were placed at the junction of two full-length capsid sequences (shown with slanted lines). The second sequence was codon-altered using synonymous substitutions to avoid homologous recombination. **(B)** Rescue and growth kinetics of recombinant ZIKV, KUNV, DENV2, and DENV4 variants. After transfection of the *in vitro* transcribed RNA, the supernatant was collected at the indicated time points, and the viral titer was quantified using an FFA. Each time point represents the average titer obtained from three independent experiments (N = 3, n = 3 replicates per experiment) with the respective standard deviations. **(C)** Left panels: localization of fluorescent reporter markers and envelope proteins. Vero cells were infected with P_0_ stocks of ZIKVmCherry and ZIKV-oxGFP at an MOI of 0.1 and with P_0_ stocks of DENV2-oxGFP, DENV2-mCherry, DENV4-mCherry, DENV4-oxGFP, KUNV-oxGFP, and KUNV-mCherry at an MOI of 0.05. At 72 h post-infection, cells were fixed and stained against viral envelope protein (green for cells infected with viruses expressing mCherry, and red for cells infected with viruses expressing oxGFP). mCherry and oxGFP signals were detected using confocal microscopy; nuclei were counterstained with Hoechst (blue). Scale bars: 10 μm. Right panels: colocalization of red and green fluorescence (Manders’ coefficient) for ZIKV-mCherry (1,082 cells), KUNV-mCherry (680 cells), DENV2-mCherry (642 cells), DENV4-mCherry (600 cells), ZIKV-oxGFP (716 cells), KUNV-oxGFP (622 cells), DENV2-oxGFP (706 cells), and DENV4-oxGFP (645 cells). One-way ANOVA followed by Tukey’s multiple comparisons test was used for statistical analysis; *p < 0.05; ns, not significant. Data are mean ± SD from three independent experiments.

To extend the study, along with the preparation of the icDNA clones containing the wt sequence of DENV2, DENV4, and KUNV, icDNAs corresponding to recombinant viruses with inserted reporter-encoding genes were also constructed. The reporters allowing the detection of the infection by either quantification of the marker expression/activity (nanoluciferase, NLuc) or visually through the fluorescence of the marker (oxGFP and mCherry) were used. It has been established that the position and insertion strategy of sequence encoding for markers in flavivirus genome play a crucial role in the genetic stability of recombinant virus and affects the speed of loss of marker over passages (Baker et al., 2020). In our study, the design previously used for the construction of stable reporter viruses of ZIKV was applied for DENV2, DENV4, and KUNV. In this design, the sequence encoding marker protein is placed between the native sequence encoding for flavivirus capsid protein and that of the foot-and-mouth disease virus (FMDV) 2A autoprotease followed by a codon-altered copy of sequence encoding for the capsid protein (Figure 1A). Thus, upon translation of the recombinant genome, the marker is released from the first copy of the capsid protein by mechanisms naturally used for the release of flavivirus capsid from the polyprotein, while FMDV 2A cleaves itself from the following copy of the capsid protein, allowing the efficient release of the reporter with no or minimal disturbance of the maturation of flavivirus structural proteins, as was confirmed by immunoblots (Supplementary Figure S1A).

### Rescue and properties of wt and reporter-expressing variants of DENV2, DENV4, ZIKV, and KUNV

The rescue of DENV2-wt, DENV4-wt, ZIKV-wt, and KUNV-wt as well as their variants encoding mCherry, oxGFP, or NLuc reporters was performed in Vero cells (Figure 1). A focus forming assay (FFA) using pan-flavivirus E protein-specific mouse MAb (4G2) was used to determine the titers of each of the rescued viruses at different days post-transfection (dpt). Supernatants designated as P_0_ stocks were collected at the peak of virus release and used to infect new cells for imaging and further analysis.

It was observed that ZIKV-wt, ZIKV-mCherry, and ZIKV-NLuc were cytopathic and showed similar exponential growth. ZIKV-wt became detectable at 2 dpt and a maximum titer of 6.5 × 10^7^ FFU/mL was reached at 7 dpt (Figure 1B). Curiously, but for unknown reasons, at early time points, ZIKV-oxGFP had titers that were approximately 20× higher than other clone-derived variants of ZIKV, reaching 5.5 × 10^9^ FFU/mL at 5 dpt; however, after that, the titer decreased, and by 8 dpt, had become comparable with the other ZIKV constructs used in the study (Figure 1B). After transfection with transcripts of ZIKV-mCherry, the first mCherry-expressing cells were observed at 4 dpt, and their number increased in the following days (Supplementary Figure S2). Interestingly, despite a high viral titer, ZIKV-oxGFP-infected cells showed a low fluorescent signal-to-noise ratio (SNR ~10), which reduced the interest in ZIKV-oxGFP as a reporter virus. By contrast, ZIKV-mCherry-infected cells showed a robust fluorescent SNR of ~40, thus increasing the value of the use of this virus as a reporter in fluorescence-based experiments, e.g., drug screening.

Cells transfected with transcripts of the icDNAs of KUNV displayed stronger cytopathic effects (CPE) than those observed for ZIKV: a complete CPE was observed already by 7 dpt. Coherently, the rescue of KUNV was also more rapid: at 2 dpt the titer of KUNV-wt was already 2.8 × 10^5^ FFU/mL and reached maximum values of 3.4 × 10^7^ FFU/mL at 4 dpt (Figure 1B). A slight delay in the development of CPE was observed for KUNV-oxGFP and KUNV-NLuc, which was reflected in reduced titers at 2 dpt. Up to 5 dpt, the titers of KUNV-mCherry were the lowest; however, at 6 dpt, they reached a level similar to those of KUNV-wt, KUNV-oxGFP, and KUNV-NLuc (Figure 1B). As an example, mCherry-reporter fluorescence was detected in viral clone-transfected cells at 4, 5, and 7 dpt up to 12 dpt depending on the virus (Supplementary Figure S2). Additionally, the replication of reporter viruses was confirmed by staining with viral envelope (E) antibodies (Figure 1C). Interestingly, fluorescent microscopy showed a distinguishable feature of recombinant KUNV replication: both mCherry and oxGFP signals were mostly detected in large intracytoplasmic structures in infected cells, reflecting the colocalization of capsids, whereas the localization of oxGFP signal in the nucleoli was observed in only a limited number of cells. Viral E proteins were associated with the plasma membrane in both cases (KUNV mCherry and oxGFP) (Figure 1C). The rescue of DENV2 and DENV4 was considerably slower than that of KUNV. Interestingly, in contrast to ZIKV and KUNV, no decrease of viral titers was observed at late time points (up to 12 dpt); instead, virus titers either reached a plateau level (as observed for DENV2-wt) or continued to increase slowly (Figure 1B). Although DENV2-wt rapidly reached high titers (approximately 1.5 × 10^7^ FFU/mL), lower titers were observed for all the reporter-harboring variants of this virus. For DENV2-oxGFP, this was observed for earlier time points and ultimately the virus reached titers similar to those of DENV2-wt. By contrast, for DENV2mCherry and DENV2-NLuc, lower titers were observed at all time points during the experiment and the maximal titer remained as low as 5 × 10^5^ FFU/mL (Figure 1B). Similar “behavior” was observed for reporter variants of DENV4; for all of these (including DENV4-oxGFP), titers remained lower than that of DENV4-wt over the entirety the experiment (Figure 1B). Similar to ZIKV-oxGFP, the fluorescent signal in DENV2-oxGFP and DENV4-oxGFP positive cells remained dim and was detected as foci (DENV2) or was diffusely distributed in the cell cytoplasm and nucleus (DENV4). For all recombinant viruses harboring mCherry or oxGFP markers, viral E protein predominantly accumulated in the cell cytoplasm (Figure 1C), where it colocalized with fluorescent marker protein. For DENV4-mCherry and DENV4-oxGFP, colocalization was at 87 and 88% (Manders’ coefficient value), respectively; similar colocalization was also observed for ZIKV and KUNV recombinants (Figure 1C). For DENV2-mCherry- and DENV4-mCherry-infected cells, the fluorescent mCherry signal was distributed within the cell cytoplasm and in the nucleus (Figure 1C). The lowest colocalization for viral E protein and the fluorescent marker was observed for DENV2-oxGFP. Most likely, this is not caused by specific properties of the virus/marker combination as estimation of the percentage of the cells positive only for oxGFP in the total population of infected cells revealed a rapid loss or inactivation of oxGFP marker (Figure 1C).

**Figure 2:**
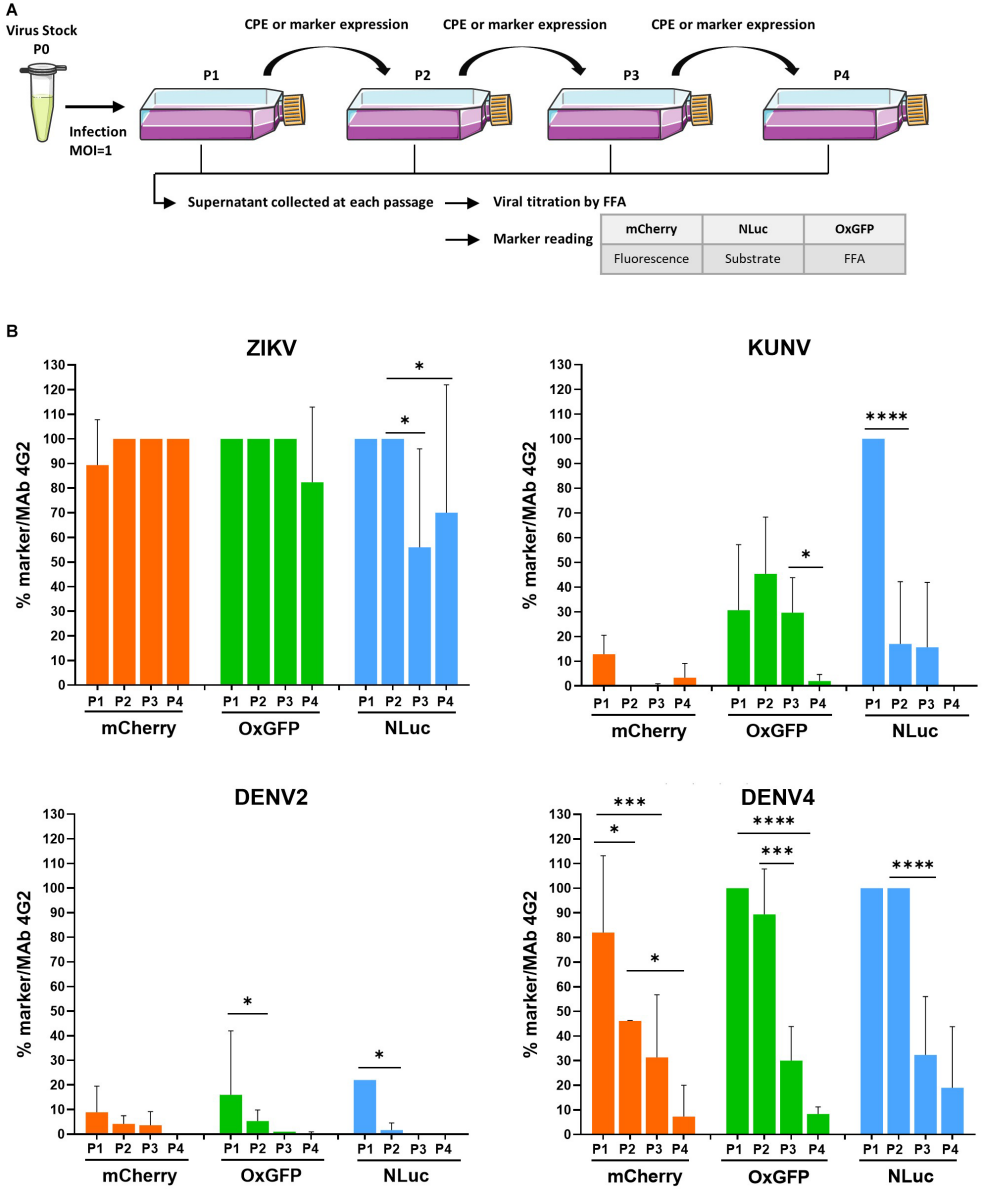
Evaluation of the genetic stability of -mCherry, -oxGFP-, and -NLuc reporter-carrying flaviviruses. **(A)** Schematic of the experimental protocol used to perform the marker stability assay. Vero cells were infected at an MOI 1 with P_0_ stocks of recombinant ZIKV, KUNV, DENV2, or DENV4 with mCherry, oxGFP, or NLuc markers and incubated until the development of CPE or visible marker expression. P_1_ stocks were collected and used to infect Vero cells for the next passage until passage 4 (P_1_ to P_4_ stocks). Viral stocks were titrated using an FFA with a mouse pan-flavivirus anti-Env antibody (Mab 4G2). mCherry signal was measured by quantifying fluorescence using a Cellomics ArrayScan VTI microscope. oxGFP expression was detected by an immunofluorescence assay using an anti-GFP antibody. For the measurement of NanoLuciferase activity, 96-well plate was infected with P_1_ to P_4_ stocks according to the end-point titration method, and NLuc was measured using the Nano-Glo Luciferase Assay System after cell lysis, on a microplate reader. **(B)** Evolution of transgene expression through serial passages for each virus. The data represent the expression of the marker reported to the infectious titer. One-way ANOVA followed by the LSD post-hoc test was used to evaluate the significance of the relationship. Each bar represents the mean ± SD (*n* = 3). *p < 0.05, ***p < 0.001, ****p < 0.0001. LSD, least significant difference.

After RT-PCR amplification (Supplementary Figure S3), we also checked by virus sequencing that no mutations were observed in the structural genes of the viruses, 7 days post-transfection of the viral stock P_0_, as compared with the WT, and in the markers of the ZIKV, DENV4, DENV2, NanoLuc, mCherry, and Ct-mCherry viruses (Supplementary Figure S4). In some instances, the markers contained a few minor silent mutations. Only, DENV2-oxGFP and KUNV-mCherry-OxGFP presented some deletions of the duplicated capsid associated with the marker or of the marker itself (see Supplementary Figures S4, S5), coherent with what was observed with the genetic stability assay (Figure 2).

Overall, the results of these experiments demonstrated the efficient rescue of recombinant flaviviruses from the RNA transcripts and revealed the dynamics of their replication, allowing us to distinguish specific phenotypic features of infection. In addition, all oxGFP- and mCherry-expressing constructs, with the exception of DENV2-oxGFP, showed a high level (Mander’s coefficient value of >80%) of colocalization coefficients between the viral E proteins and their respective markers (Figure 1C). As expected, the replication of ZIKV-wt, KUNV-wt, DENV2-wt, and DENV4-wt was (with the notable exception of ZIKV-oxGFP, which we could not explain reasonably) more robust than that of viruses containing genes encoding for fluorescent or NLuc markers. These data indicate that the insertion of the marker sequences had an impact on the level of RNA replication and/or virion formation and release.

### Assessment of the genetic stability of recombinant virus genomes in cell culture

Flaviviruses carrying reporters are known to face instability problems. Owing to mutations and recombination events, the reporter genes are often lost during the serial passaging of these viruses. The stability of recombinant flavivirus genome depends on multiple factors, including virus species, the marker insertion strategy, and the marker gene used (Baker and Shi, 2020). Once the marker is lost, the resulting virus will eventually overgrow the parental recombinant; the speed by which the virus outcompetes the parental recombinant depends on the conditions of infection (MOI) and differences in the growth kinetics of the competing viruses. All marker-containing viruses analyzed in previous experiments, except for ZIKV, replicated slower than their wt counterparts (Figure 1B), demonstrating a growth advantage of the wt virus and indicating that viruses that have lost a sequence encoding for a marker very likely also have a growth advantage. Therefore, we analyzed the marker stability of all the aforementioned viruses with reporters by performing four passages in Vero cells and subsequently quantifying the infectious titer using FFA (immunostaining against E) and analyzing marker expression by fluorescence microscopy (mCherry), fluorescence microscopy with immunostaining (anti-GFP), or measuring luminescence (NLuc) (Figure 2A).

Coherent with a previous study (Mutso et al., 2017), all recombinant variants of ZIKV displayed relatively stable marker expression over four passages, with ≥80% cells infected with P_4_ stock of ZIKV-mCherry- or ZIKV-oxGFP (Figure 2B). Results obtained for ZIKV-NLuc showed a larger variation, indicating a partial loss of the inserted sequence during late passages; however, approximately 50% of cells infected with P_3_ or P_4_ stocks did express NLuc (Figure 2B), as measured by TCID50 using both FFA and direct NLuc measurement. By sharp contrast, a loss of marker expression was already detected for P_1_ of KUNV-mCherry and KUNV-oxGFP; cells infected with P_2_ of these viruses revealed almost complete (mCherry) or > 50% (oxGFP) loss of marker expression (monitored by FFA with 4G2 antibody labeling). Marker expression was detected in all cells infected by P_1_ of KUNV-NLuc; however, in subsequent passages, the percentage of cells infected with the viruses expressing the marker diminished, and by passage 4, marker expression was completely lost (Figure 2B). The effect was similar, albeit even more pronounced, for all recombinant DENV2 variants. Approximately 90% of the loss of marker expression occurred during the first passage, and marker expression was not observed during subsequent passaging (Figure 2B). Somewhat surprisingly, DENV4 reporter-harboring variants were clearly more stable. mCherry expression was almost uniformly detected in cells infected with P_1_ of DENV4mCherry followed by a gradual decrease of marker-positive-infected cells over the next three passages. Both DENV4-oxGFP and-NLuc viruses were stable for two passages; however, a rapid loss of markers was observed for P_3_ and P_4_ stocks of these viruses (Figure 2B).

### DENV2-mCherry can be stabilized by truncating the sequence encoding for the first copy of capsid protein

**Figure 3:**
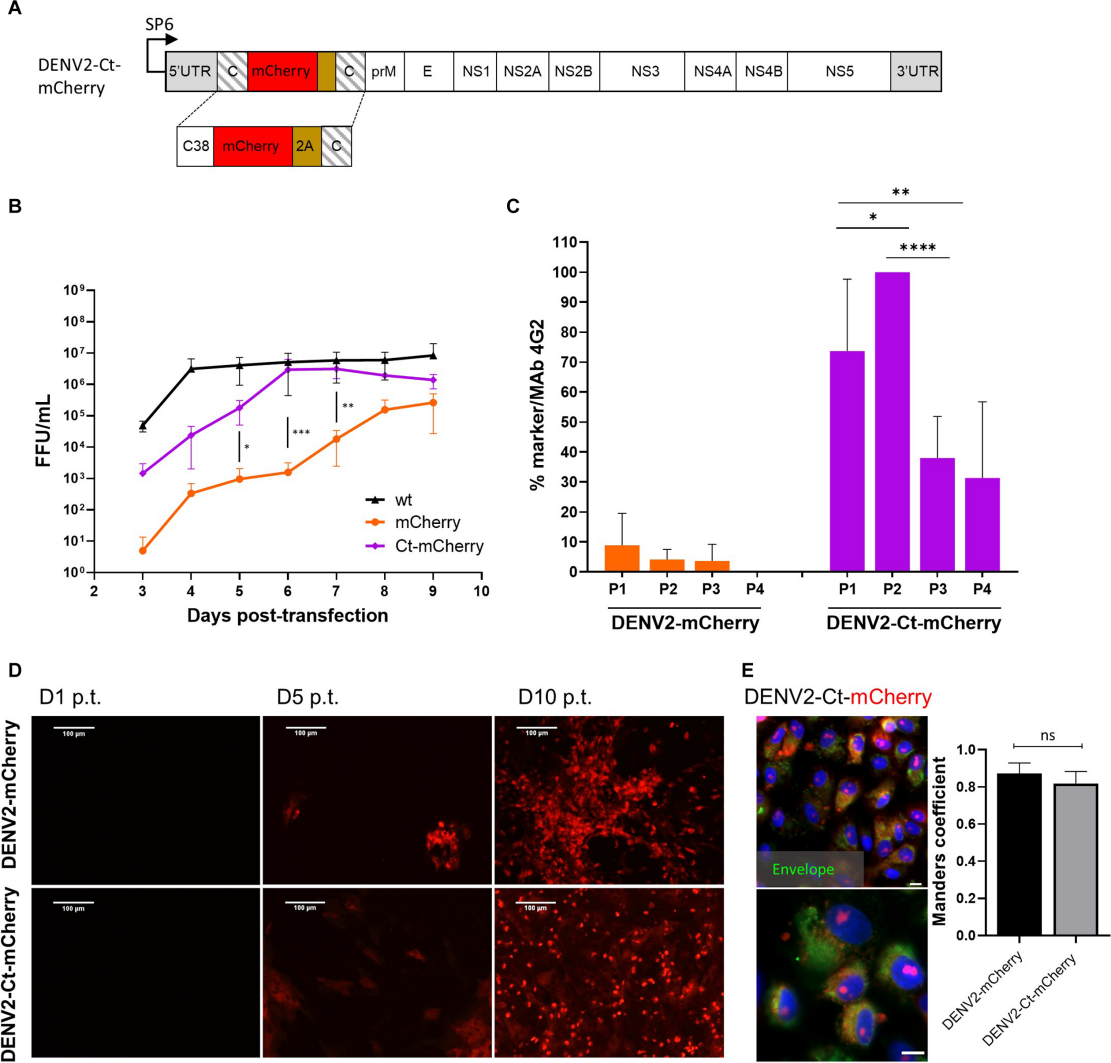
Comparison of the growth kinetics and genetic stability of DENV2-mCherry and DENV-Ct-mCherry. **(A)** Schematic representation of the DENV2-Ct-mCherry icDNA clone. The first copy of the capsid protein was truncated by removing residues 39–114. The rest of the design corresponds to the icDNA clones of viruses with full-length capsid duplication. **(B)** Growth kinetics of recombinant DENV2-wt, DENV2-mCherry, and DENV2-Ct-mCherry. Standard deviation is calculated from replicate (*N* = 3; *n* = 3 replicates per experiment) samples tested at each time point. One-way ANOVA was used to evaluate the significance between mCherry and Ct-mCherry clones. Each bar represents the mean ± SD (*n* = 3). **(C)** Comparison of the marker stability of recombinant DENV2-mCherry and DENV2-Ct-mCherry over four passages. One-way ANOVA followed by a least significant difference post-hoc test was used to evaluate the significance of the relationship. Each bar represents the mean ± SD (*n* = 3). **(D)** Evaluation of replication dynamic DENV2-mCherry or DENV2-Ct-mCherry based on the increasing intensity of mCherry signal in the virus-infected cells over the indicated time points. **(E)** Left panels: confocal images of mCherry fluorescence (red) and DENV2 envelope protein (green) at 72 h p.i. at an MOI of 0.05 (scale bars: 50 μm and 10 μm, respectively). Right panel: colocalization of red and green fluorescent signals (Manders’ coefficient) for DENV2-mCherry (642 cells) and DENV2-Ct-mCherry (616 cells). Data represent mean ± standard error of mean (*n* = 3). ANOVA was performed followed by Tukey’s multiple comparisons test. *p < 0.05; **p < 0.01; ***p < 0.001; ****p < 0.0001; ns, not significant.

The instability of reporter-expressing recombinant flaviviruses has led to the development of various approaches aimed to overcome the problem. For viruses harboring markers between two copies of sequences encoding for capsid protein (Figure 1A), shortening of the first (native) copy of the capsid protein gene to a length sufficient for preserving *cis-*active RNA elements located in this region has been successfully used; sometimes, such truncation is combined with additional modifications of the insertion region (Baker et al., 2020; Volkova et al., 2020). For some flaviviruses, the optimal length of the 5′ copy of the capsid encoding sequence has been determined to be 35 or 38 codons; more extensive truncations have been shown to cause unpredictable recombinations of the genome or the loss of the marker (Samsa et al., 2012; Baker et al., 2020). To determine whether this approach can be used to stabilize highly unstable DENV2-mCherry, residues 39–114 in the first copy of capsid were deleted (Figure 3A). The rescue and properties of the corresponding virus (designated as DENV2-Ct-mCherry) were compared with those of DENV2-mCherry (Figures 3B,C).

Higher titers were obtained for DENV2-Ct-mCherry over the time course of the virus rescue experiment than for DENV2-mCherry. By 6 dpt, the titers of DENV2-Ct-mCherry reached a plateau level (around 5 × 10^6^ FFU/mL) that was close to that of DENV2-wt (Figure 3B). Remarkably, improved genetic stability was also observed: all or nearly all cells infected with P_1_ or P_2_ stocks of DENV2-Ct-mCherry were positive for mCherry expression; in cultures infected with P_3_ or P_4_ stocks, the loss of the marker became more evident yet 30 to 40% of infected cells were still positive for mCherry (Figure 3C). No clear difference in the time scale of detection of mCherry fluorescence in reporter-positive cells was observed between DENV2mCherry and DENV2-Ct-mCherry (Figure 3D). In both cases, fluorescent signals were detected in the nucleus and cell cytoplasm, with Manders’ coefficients of 0.87 and 0.82 for DENV2-mCherry and DENV2-Ct-mCherry, respectively (Figure 3E). The nuclear accumulation of mCherry has also been reported for other DENV2 constructs of a similar design and was most likely caused by the fusion of mCherry with capsid protein or its fragment (Bulich and Aaskov, 1992; Sangiambut et al., 2008; Netsawang et al., 2010). Previously published data indicate that the appearance of the capsid protein in the nucleus of DENV2-infected cells occurs due to the presence of a nuclear localization signal that facilitates interaction with cellular proteins responsible for nuclear transport in its sequence (Sangiambut et al., 2008; Zhang et al., 2021). Taken together, truncation of the first copy of the capsid protein gene had a positive impact on the replication and genetic stability of DENV2 harboring the mCherry marker without any apparent changes in the subcellular distribution of the reporter. Overall, the same approach, i.e., a truncated capsid, can be used to develop flavivirus infectious clones harboring reporters to overcome issues related to the genetic instability of the corresponding recombinant viruses.

### Imaging of recombinant virus-infected cells by TEM and purified viruses by Bio-AFM

**Figure 4:**
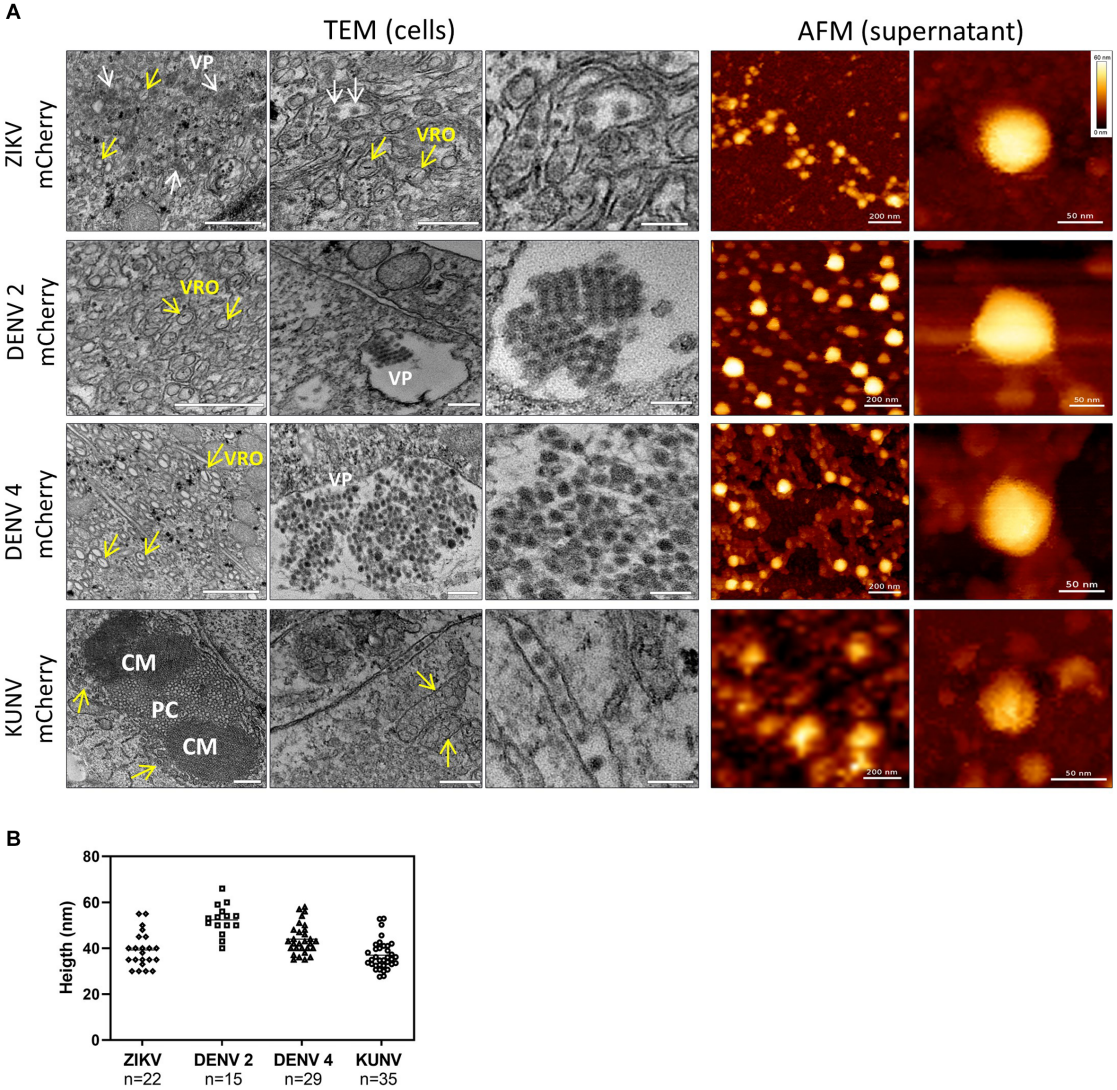
TEM and AFM images of mCherry-reporter virions. (**A**, left panel) Transmission electron microscopy of cells infected with DENV2-mCherry, DENV4-mCherry, ZIKV-mCherry, and KUNV-mCherry. Replication organelles derived from the ER were visualized. CM, convoluted membranes; PC, paracrystalline arrays; VP, vesicle packets (white arrows). The localization of viral replication organelles (VRO) are shown with yellow arrows. Scale bars = 200 nm (left and central panels) and 100 nm (right panels). (**A**, right panel) AFM images of mCherry-expressing ZIKV, DENV2, DENV4, and KUNV virions purified from cell supernatants. Virions of ZIKV-mCherry were purified by dialysis in PBS, virions of DENV2mCherry and DENV4-mCherry were purified by Microsep advanced column from the Pall Corporation in Tris-NaCl buffer, and virions of KUNV-mCherry were purified by ultracentrifugation on a sucrose cushion. Scale bars = 200 nm (left panels) and 50 nm (right panels). **(B)** Distribution of the topographical maximal height of viral particles (ZIKV, *n* = 22; DENV2, *n* = 15; DENV4, *n* = 29; and KUNV, *n* = 35) measured from cross-sections of AFM images.

To image the morphogenesis of virions of clone-derived mCherry-expressing viruses, we performed a cross-analysis of resin-embedded Vero cells infected at an MOI of 1 by P_0_ stocks at 3 days post-infection by transmission electron microscopy (TEM). The replication of flaviviruses typically causes dramatic remodeling of the endoplasmic reticulum (ER) membranes, which wrap around the viral replication factories to form viral replication organelles (VRO) (Mackenzie et al., 1996), convoluted membranes/paracrystalline arrays (CMs/PC) (Caldas et al., 2020), and vesicle packets (VP) used as loci for viral genome amplification, RNA translation, and polyprotein processing (Stollar et al., 1967; Westaway et al., 1997; Mackenzie et al., 2001; Welsch et al., 2009; Hamel et al., 2015; Barreto-Vieira et al., 2017; Aktepe and Mackenzie, 2018). Coherently, the infection of Vero cells with ZIKV-mCherry, DENV2-mCherry, DENV4-mCherry, or KUNV-mCherry induced massive ultrastructural ER expansions and reconfigurations, cytoplasm vacuolization, the formation of ER sheets and ER-derived vesicles containing electron-dense viroplasm-like structures, and newly formed virions (Figure 4A, left panels). CMs were usually found in the center of large structures (Figure 4A), as has been previously observed in cells infected with DENV or ZIKV (Welsch et al., 2009; Caldas et al., 2020). VRO and VP were unambiguously observed within subcompartments, including the lumen of large cytoplasmic vacuoles, with a dramatic accumulation of PC and membrane-associated virus particles, often arranged in regular arrays for DENV2- and KUNV-infected cells (Westaway et al., 1997; Mackenzie et al., 2001; Aktepe and Mackenzie, 2018). Tubular-altered ER containing immature viral particles were also observed in close proximity to the VP (Figure 4). All these features have been commonly observed in mammalian cells producing flaviviruses (Mackenzie et al. 1996; Caldas et al., 2020).

Next, Bio-AFM was used to analyze the morphology and integrity of virions released from cells infected with ZIKV-mCherry, DENV2-mCherry, DENV4-mCherry, or KUNV-mCherry. Viral particles were purified from cell supernatants, resuspended/diluted in a buffer solution, and smoothly adsorbed on a poly-L-lysine-coated mica surface. To preserve the structural integrity of the Bio-AFM imaging was performed in a buffer, using a Bio-AFM operating in a BSL3 environment. Figure 4 (right panels) shows topographic AFM images of purified viruses with different magnifications. Interestingly, in all cases, fractions of the viral particles appeared to be clustered, reminiscent of the viral clusters observed in the ER lumen by TEM, which suggests that the virions can be released from the infected culture cells as viral “packages” or, for KUNV-mCherry, can be clustered during ultracentrifugation. Individual virions of ZIKV-mCherry, KUNV-mCherry, DENV2-mCherry, and DENV4-mCherry appeared as roughly spherical particles with some angles suggesting an icosahedral arrangement, as expected from their reported cryoEM structures (Ferreira et al., 2008; Kostyuchenko et al., 2014;Sirohi et al., 2016; Sevvana et al., 2018). Viral particle height measurements obtained from cross-section analysis (Figure 4B) were also consistent with the virion sizes measured by cryoEM or previous AFM studies: 52 ± 8 nm for DENV2-mCherry (Ferreira et al., 2008), 44 ± 7 nm for DENV4-mCherry (Kostyuchenko et al., 2014), 39 ± 9 nm for ZIKV-mCherry (Sirohi et al., 2016; Sevvana et al., 2018), and 37 ± 8 nm for KUNV-mCherry (Therkelsen et al., 2018). DENV4-mCherry shows morphology similar to DENV2-mCherry, with a slightly smaller mean diameter. These results suggest that modification of the viral genome (adding an extra copy of sequence encoding for capsid protein and a sequence encoding mCherry, i.e., making the genome 10% larger) had no detectable impact on virion size or morphology.

### Validation of fluorescent-reporter flaviviruses for antiviral screening

**Figure 5:**
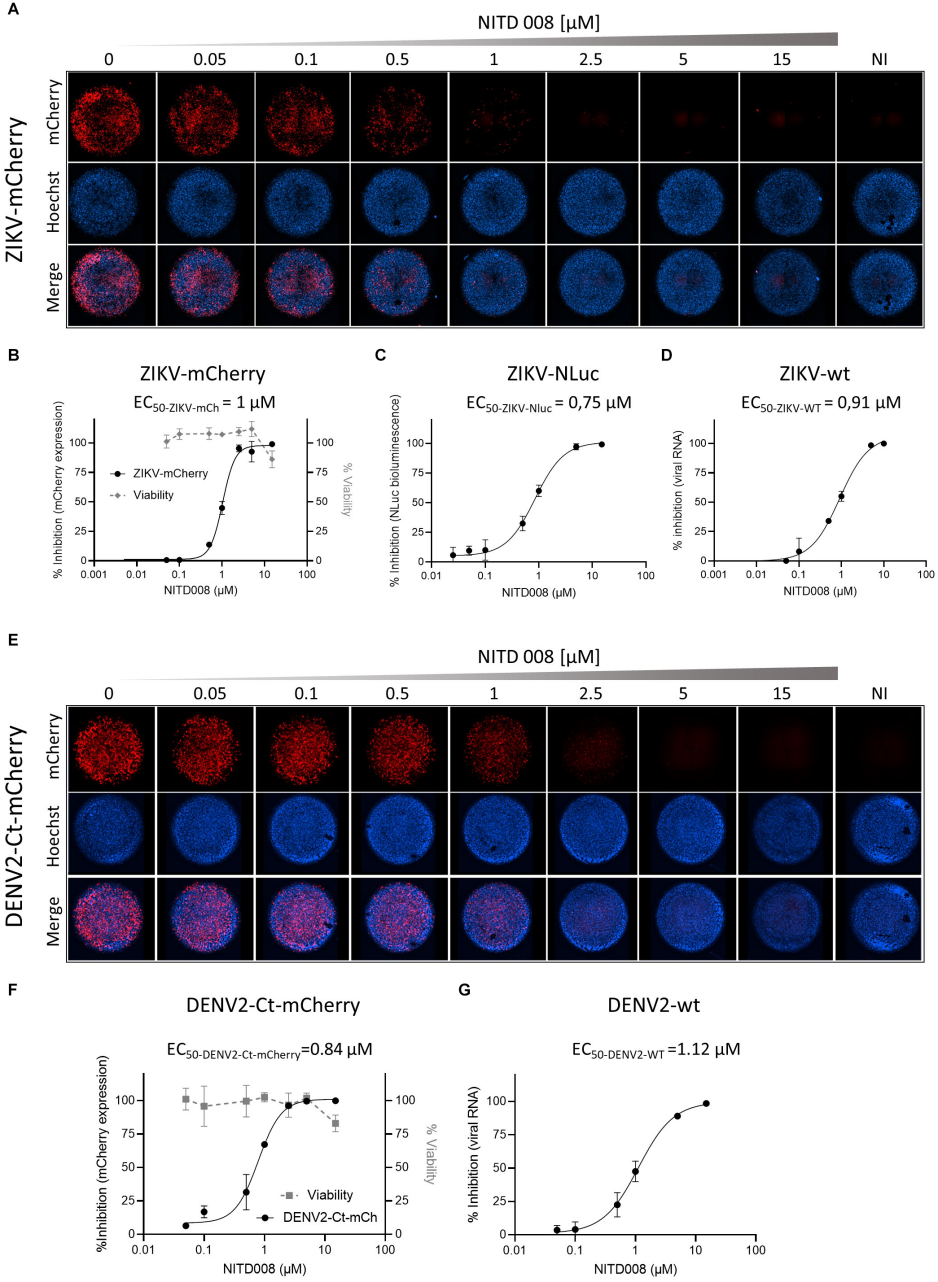
Antiviral activity of NITD008 on the reporter-expressing flaviviruses. **(A)** The dose response of NITD008 against ZIKV-mCherry was monitored by fluorescence microscopy. Each panel corresponds to a mosaic image (six tiles) of a single well within a 96-well plate with Vero cells incubated with the indicated increasing concentrations (0.05, 0.1, 0.5, 1, 2.5, 5, and 15 μM) of NITD008 and infected with ZIKV-mCherry at an MOI of 0.05. Upper line, mCherry fluorescence; middle line, cell nuclei stained with Hoechst; bottom line, merged channels. Cell viability at 3 d.p.i. against NITD008 is shown in **(B)**. Dose–response curves for ZIKV-mCherry **(B)**, ZIKV-NLuc **(C)**, and ZIKV-wt **(D)**. **(E)** Dose–response of NIT008 against DENV2-Ct-mCherry monitored by quantitative fluorescence microscopy. Vero cells were treated with increasing concentrations of NITD008 (0.05, 0.1, 0.5, 1, 2.5, 5, and 15 μM) and infected at an MOI of 0.1. Cell viability at 3 d.p.i. against NITD008 is shown in **(F)**. Dose–response curves of NITD008 are shown for DENV2-Ct-mCherry **(F)** and DENV2-wt **(G)**. The corresponding EC_50_ values are indicated in each panel.

To assess whether the fluorescent (or luminescent) reporter-expressing recombinant flaviviruses could be applied to high-throughput screening for antivirals in a two-dimensional cell culture system, we chose to test the adenosine nucleoside analog NITD008, previously shown to inhibit the replication of mosquito- and tick-borne flaviviruses, including WNV, DENV, YFV, ZIKV, and TBEV (Yin et al., 2009; Deng et al., 2016). Cells were incubated for 2 h with increasing concentrations of NITD008 and then infected with the corresponding P_0_ stocks of the reporter viruses stably expressing inserted marker protein: ZIKV-mCherry, ZIKV-NLuc, or DENV2-Ct-mCherry; ZIKV-wt- or DENV2-wt-infected cells were used for comparison (Figure 5). For ZIKV-mCherry and DENV2-Ct-mCherry, mCherry fluorescence intensity in infected cells was quantified directly on fixed cells by fluorescence microscopy, using nuclei counting for data normalization (Figures 5A,E). For a sake of comparison, cells infected with wt viruses were lysed and total viral RNA was extracted and quantified by RT-qPCR (Figures 5B,D). For ZIKV-NLuc, bioluminescence in lysates of infected cells was quantified by conducting a corresponding enzymatic assay (Figure 5C). The dose–response curves obtained allowed the extraction of the concentrations of NITD008 that inhibited 50% of viral infection (EC_50_). In accordance with previously reported data (Yin et al., 2009; Deng et al., 2016), NITD008 showed, in all cases, a similar dose-dependent inhibition of virus replication, with EC_50_ values ranging from 0.75 to 1 μM for ZIKV-mCherry and ZIKV-NLuc (Figures 5B,C) and an EC_50_ value of 0.84 μM for DENV2-Ct-mCherry (Figure 5F). Importantly, similar EC_50_ values were obtained for wt viruses with the use of RT-qPCR quantification (Figures 5D,G). These results indicate that ZIKV-mCherry, as well as ZIKV-NLuc and DENV2-Ct-mCherry, can serve as reliable tools for rapid and more direct high-throughput antiviral screening.

## Discussion

Over the last decades, reverse genetic approaches have allowed advances in the study of different aspects of flavivirus replication. With these approaches, the construction and manipulation of icDNA clones is essential; however, in contrast to many viruses, the construction and use of these tools for flavivirus studies remain a difficult task due to the instability of the plasmids containing flavivirus cDNAs (Mishin et al., 2001; Aubry et al., 2015). Despite the numerous advantages of high copy number plasmids, they were generally found to be unsuitable for the cloning of flavivirus icDNA as the presence of cryptic prokaryotic promoters in viral sequences leads to the synthesis of mRNAs encoding viral proteins that are toxic to bacteria (Pu et al., 2011). An alternative approach, the use of a low copy number plasmids, can often overcome the instability problem of the virus cDNA by lowering the level of cryptic protein expression and consequently decreasing their cytotoxicity to *E. coli* (Aubry et al., 2015). This strategy was successfully applied to obtain icDNAs for numerous flaviviruses, including YFV, KUNV, ZIKV, and DENV1, 2, and 4 (Santos et al., 2015; Shan et al., 2016; Hu et al., 2022), with DENV3 excluded because the full-length cDNA of this virus appeared to be too toxic to maintain in *E. coli* as a single unit (Santos et al., 2014). Our data reported above are in line with these studies as we could obtain single plasmid icDNA clones for ZIKV, KUNV, DENV2, and DENV4 but not for DENV3. In light of this, additional approaches to resolve the stability problem have been developed. For instance, bacterium-free methods, in particular CPER, have been successfully applied for the development of KUNV icDNA; alternatively, plasmids containing flavivirus icDNAs have been stabilized by the insertion of the intron into virus-derived sequences (Pu et al., 2011; Edmonds et al., 2013; Ávila-Pérez et al., 2018).

The use of icDNAs allows the easy modification of flavivirus genomes; however, on their own, they do not permit the visualization of the course and/or dynamic of the virus infection. A simple concept based on the insertion of the reporter gene(s) into the viral genome allowed us to overcome such a limitation. If successfully applied, recombinant viruses represent a valuable system for tracking and quantifying flavivirus replication *in vitro* and, potentially, *in vivo*. The findings obtained in the current study confirmed that such viral constructs can be used for high-content antiviral screening as well as for the acceleration discovery of antiviral molecules. However, the generation of the reporter viruses containing oxGFP, mCherry, or NLuc markers does encounter another problem—the genetic instability of rescued recombinant viruses observed as a loss of the reporters due to the recombination events during virus replication and the selection for faster-replicating recombinants (lacking a reporter gene) during virus propagation and passaging.

With regard to ZIKV-oxGFP rapid replication growth (Figure 1), viral sequencing of the regions from the beginning of the capsid protein to the end of NS1, including inserted marker sequences, did not reveal any mutations in the viral or inserted sequences. Of note, sequencing of the same region for other variants of ZIKV, as well as those of other variants of ZIKV, DENV2, DENV4, and KUNV, did not detect any mutations in the viral structural proteins of the NS1 region, indicating that such changes are either rare or/and unfavorable for recombinant viruses (see Supplementary Figures S3–S5). Consequently, the accelerated growth of ZIKV-oxGFP was not due to mutations in viral structural proteins or re-arrangements in the inserted marker region. The exact reason(s) for this phenomenon remain unknown and their analysis was beyond the scope of the current study. It can be speculated that accelerated growth may originate from some properties of oxGFP and/or is specific to the experimental conditions (cell line, growth media, etc.) used in this study, as a similar effect has not been observed previously for ZIKV harboring an eGFP insertion (Mutso et al., 2017).

Regarding the loss of the inserted sequences for some of the viruses, the sequencing of regions from the start of the coding region to the end of the NS1 gene revealed truncations, including the deletion of the inserted marker for Denv2-oxGFP, KUNV-mCherry, and -oxGFP, and in some cases, point mutations in the inserted marker gene were detected (see Supplementary Figures S4, S5). These changes are likely responsible for the loss of marker activity (as shown in Figure 2). Interestingly, in several cases, no mutations that can be attributed to the loss of the marker expression was detected. Most likely, this indicates that a large proportion of viral progeny maintained the marker but the corresponding viruses had reduced infectivity and/or fitness and were rapidly outcompeted in subsequent passages. Additionally, our data suggest that monitoring of the expression of the inserted marker is a relevant and effective method for analyzing the stability/infectivity/fitness of flaviviruses harboring recombinant genomes.

Here, we observed that recombinant flaviviruses harboring an NLuc marker were somewhat typically more stable than the ones carrying mCherry or oxGFP markers (Figure 2). Presumably, this was caused by the smaller size of the NLuc insertion (the sequence encoding NLuc is 513 bp long, whereas that for mCherry is 708 bp long). This is consistent with our previous studies with ZIKV, in which the presence of an additional 228 bp insertion encoding ubiquitin resulted in the marked destabilization of viruses encoding for mCherry or GFP reporters (Mutso et al., 2017). Such an effect was not observed for ZIKV encoding NLuc, suggesting the existence of a rather well-defined upper limit for the size of the insert that is tolerated by ZIKV. The data presented in this study suggest that this limit is different for each flavivirus and likely even for sub-types of virus, as observed with DENV2 and DENV4. Interestingly, the stability assay revealed that fluorescent markers were better tolerated by ZIKV than other tested flaviviruses (Figure 2). We observed that previously reported data about the stability of markers in ZIKV-RLuc (Shan et al., 2016), DENV2-mCherry (Li et al., 2020), and DENV2-GFP (Suphatrakul et al., 2018) is consistent with our data. Thus, the findings obtained in the current study clearly underline the high level of instability of all DENV2 and KUNV variants harboring reporters (Figure 2).

It can be speculated that depending on the viral species, genome lengthening by insertions may have a different negative impact on the cyclization of the genome and/or packaging of the viral RNA into the capsid without having a detrimental effect on the virulence of the virus. Of note, one recent discovery demonstrated that JEV had the ability to pack genomic RNA that was much larger in length (up to 15 kb) but the increased genome length was accompanied by a decreased RNA replication efficiency (Yun et al., 2007). Our data indicate that the absolute speed of the replication is not likely the cause, as KUNV variants replicate faster than those of ZIKV or DENV4 (Figure 1). In a recent study, a comparison of the stability of an NLuc insertion for 10 passages was performed for DENV1-4, ZIKV, YFV, and JEV. This study revealed a clear correlation between marker stability and the length of the 5′ copy of the capsid-encoding sequence (Baker and Shi, 2020). Our observation that a reduction in the length of the 5′ copy of the capsid-encoding sequence in DENV2-mCherry has a clear positive impact on the stability of marker expression over passages (Figure 3) is coherent with this data.

Earlier reports also confirmed that some viruses may tolerate less than 10% of an increase in their genome size during packaging into viral capsids, highlighting the importance of the relationship between the size of the viral genome and capsid formation. In this regard, it should be mentioned that we could not find any correlation between the size of the RNA genome and recombinant reporter virus stability, as the sizes of the ZIKV (10,807 nt), KUNV (11,022 nt), DENV2 (10,723 nt), and DENV4 (10,649 nt) genomes are similar. It may be the ratio of the size of the genome to the size of the capsid or the virion that is important—not all viruses may have the same capsid size and the compaction of the viral genome would be higher in a smaller capsid. Clearly, more detailed studies are needed to precisely discover the mechanism(s) responsible for the disadvantage (and therefore counterselection) of flaviviruses with increased genomic RNA lengths.

Capsid protein is known to be a central element in the flavivirus assembly process, which due to the precise physical interactions with the viral genome stabilize the capsid (Tan et al., 2020). The anchor domain (α5 helix) of the capsid possesses a key function in the virion assembly, and in case of its absence, the capsid dimers may remain “locked” within the RNA core, impeding the correct formation of the virions. What is more interesting, the controlled and timely manner of the capsid α5 helix processing was shown as one of the crucial factors necessary for incorporating the nucleocapsid into the virions. Subsequently, α5 helix is removed from the capsid protein but recent investigations demonstrated the opposite for ZIKV: α5 helix is retained for some of the capsid subunits present in virions, indicating other possible (additional) functions in the assembly process (Tan et al., 2020; Barnard et al., 2021). Additionally, the threshold of the positive charges localized in the N-terminal part of the capsid protein, which are necessary for the correct formation of the virions, may be different and thus may affect the stability of flaviviruses to a different extent. Taken together, requirements for the involvement of the capsid protein in the coordination of virion formation are different, which in turn may influence the overall stability of the increased length of the genome or its packaging. Moreover, the functional properties of the RNA elements present within the first 38 codons in the 5′ copy of the capsid gene were different among flaviviruses (Volkova et al., 2020). This indicates that the shortening of this sequence may not affect viral fitness to the same extent for all flaviviruses.

To date, *Flavivirus* particles sizes for ZIKV (49 nm) (Sirohi et al., 2016), DENV2 (50 nm) (Kuhn et al., 2002; Zhang et al., 2003), DENV4 (48 nm) (Kostyuchenko et al., 2014), and KUNV (50 nm) (Therkelsen et al., 2018) have been determined by cryoEM. The AFM measurements performed during this study (Figure 4) with reporter viruses containing an mCherry insertion in their genomes are consistent for DENV2 (52 nm) and DENV4 (44 nm) particle sizes; interestingly, somewhat smaller particle sizes were obtained for ZIKV (40 nm) and KUNV (37 nm). This suggests a slight deformation of ZIKV and KUNV particles during the AFM experiment that could be due either to their adsorption on the surface or the force applied by the AFM tip (or both). On the other hand, DENV2 and DENV4 are maybe more structurally stable in these conditions, showing comparable particle sizes between cryoEM and AFM imaging. However, our results suggest an absence of correlation between particle size and marker stability, as exemplified by ZIKV. Therefore, the size of the virion may be affected—to some extent—by the envelope organization rather than the capsid/genomic RNA size. This leaves the question open. In addition, packages of secreted virions (Figure 4) have not been described so far, to our knowledge, and further exploration is required to attribute this phenomenon to particle preparation for imaging or to a biological behavior.

How does the loss of marker occur? Here, we did not analyze the mechanism directly but some conclusions can be based on the observed speed of the marker loss. Previous studies indicate that the initial loss of the marker occurs through in-frame deletions in the reporter encoding sequence. In most cases, the loss of reporter did not occur (or, at least did not become detectable) during the rescue; instead, it occurred, often very rapidly, during passaging of the rescued virus. It is thus very likely that the main mechanism of how markerless viruses become dominant is not the loss of the marker itself (the timing or frequency of such an event) during viral rescue and replication. Instead, our data is more coherent with the hypothesis that the difference between the growth kinetics of wt virus and virus with a reporter may be the key: the smaller the growth advantage of the wt virus the more stable its variants are with a reporter. The significant growth advantage of wt viruses most likely extends to viruses harboring a deletion in the marker region and allows them to rapidly outcompete the ones that have maintained a marker.

Given these considerations, new approaches directed at increasing the stability of recombinant flavivirus genomes that preserve the properties and virulence similar to the wild-type virus are needed. To date, several strategies have been developed and tested. One of them is the utilization of recombination-dependent lethal mutations. The introduction of such a change into the capsid region of ZIKV and YFV is capable of increasing stability and preventing the formation of defective flavivirus virion in case of recombination events. At the same time, the use of split reporter proteins can also be considered to be one of the most promising approaches due to their already proven robustness and effectiveness. Finally, a recent study has shown that a point nucleotide mutation (T142C) in the 5’ CS of JEV, WNV, and DENV leads to the mutation of the capsid protein at position 16 (M16T), which in turn assists genome cyclization with the following stabilization of the reporter-harboring viruses (Li et al., 2017). The question that remains open is whether the combination of several previously mentioned approaches can increase the stabilizing effect.

## Materials and methods

### Cell culture

Vero cells (African green monkey kidney cells, ATCC CCL-81) were grown in Dulbecco’s modified Eagle’s medium (DMEM, Lonza) supplemented with 10% fetal bovine serum (FBS, Gibco), 100 U/mL of penicillin, and 100 μg/mL of streptomycin at 37°C with 5% CO_2_.

### Design and assembly of wt and reporter harboring icDNA clones of flaviviruses

The construction of an icDNA clone of ZIKV (Brazilian isolate), designated as ZIKV-wt, and its variants harboring mCherry and NLuc reporters (ZIKV-mCherry and ZIKV-NLuc), has been described previously (Zheng et al., 2016). To obtain ZIKV expressing green fluorescent marker, NLuc was replaced with an oxidation-resistant GFP marker (McGee et al., 2010); the obtained clone was designated ZIKV-oxGFP. The same cloning strategy was applied for the construction of icDNA clones of DENV2, DENV4, and KUNV. Briefly, synthetic DNA fragments were obtained from Twist Bioscience (San Francisco, CA, USA) and Genscript (New Jersey, USA). The assembly strategy included five steps in which the synthetic fragments were consequentially cloned into the single copy pCCI-Bac plasmid with the SP6 promoter placed upstream of the region corresponding to the 5′ end of the virus genome. The oxGFP, mCherry, and NLuc marker genes were cloned between two copies of capsid sequences in the structural region of the modified genome, as described previously (Zheng et al., 2016). The obtained clones were designated as DENV2-wt (-oxGFP, -mCherry, and -NLuc), DENV4-wt (-oxGFP, -mCherry, and -NLuc), and KUNV-wt (-oxGFP, -mCherry, and -NLuc) (see Supplementary Table S1 for the genomic sequences). To reduce the loss of the mCherry marker, a 228 bp deletion, removing codons 39–114 of the first (native) copy of the region encoding for the capsid protein of DENV2, was introduced into DENV2-mCherry using PCR-based mutagenesis and subcloning procedures; the resulting clone was designated as DENV2-Ct-mCherry. Cloning procedures and amplification of the obtained plasmids containing the icDNAs of the viruses were carried out in *E. coli* EPI300 cells (LGC Biosearch Technologies, United Kingdom). Sequences of all the obtained plasmids were confirmed using Sanger sequencing and are available from the authors upon request.

### *In vitro* transcription and virus rescue

icDNA plasmids (10 μg) of DENV2-wt, DENV4-wt, ZIKV-wt, KUNV-wt, and their reporter-containing variants were linearized using AgeI-HF enzyme (NEB, United States) prior to *in vitro* transcription. The linearized DNAs were purified using a Monarch DNA cleanup kit (NEB, United States) and the capped RNA transcripts were synthesized using an SP6 mMessage mMachine kit (Invitrogen, United States) following the manufacturer’s instructions. All virus studies were conducted in a biosafety level 3 facility in CNRS CEMIPAI Montpellier. Vero cells were transfected with the obtained RNA transcripts using Lipofectamine 2000 (Invitrogen) reagent. Briefly, transfection mixtures were incubated at room temperature for 5 min and added to Vero cell monolayers and then incubated for 5–6 h at 37°C. The cells were then washed in 1 × PBS (Eurobio, France) and incubated in growth medium at 37°C. Supernatants (P_0_ stocks) were collected at 7 to 15 days post-transfection, clarified by centrifugation at 1,000 × g for 10 min, aliquoted, and stored at −80°C.

### Focus forming assay

Viral supernatants were titrated using the end-point titration method. Vero cells were seeded on 96-well plates at 10,000 cells per well and incubated for 4 h at 37°C. Cells were infected with virus dilutions (from 10^−1^ to 10^−9^) prepared in DMEM supplemented with 2% FBS and a 1% penicillin/streptomycin mixture (Lonza Biosciences). Infected cells were incubated at 37°C for 7–15 days. The medium was then removed, cells were washed once with PBS, and fixed with 4% paraformaldehyde (PFA, Fisher) for 30 min at room temperature. After the removal of PFA, cells were washed with PBS and kept at 4°C until staining. For the staining, cells were permeabilized with PBS containing 0.1% Triton X-100 (Sigma-Aldrich, France) for 5 min and blocked with PBS containing 2% FBS and 0.05% Tween 20 (Sigma-Aldrich, France) for 1 h. Cells were washed once with PBS and incubated with a mouse pan-flavivirus anti-Env antibody (Mab 4G2, Novus Biologicals NBP2) for 1–2 h at room temperature (1:1,000) for viral titration or an anti-GFP antibody (A11122, Invitrogen) for the measurement of oxGFP-positive cells. Following this, cells were washed 3 times for 10 min each time with PBS containing 0.1% Tween 20 and then incubated in the dark with a fluorescent goat anti-mouse IgG secondary antibody conjugated with DyLight 800 (Invitrogen, SA5-35521) for 1–2 h at room temperature. Finally, cells were washed 3 times for 10 min each time with PBS containing 0.1% Tween 20 and the fluorescence was recorded using a microplate reader (Odyssey, Li-Cor Biosciences, United States). Viral titers were calculated using the Spearman–Kärber algorithm.

### Genetic stability assay

Vero cells were seeded on 6-well plate at 300,000 cells per well. Cells were infected at an MOI of 1 with P_0_ supernatants corresponding to each of the rescued marker expressing viruses. After incubation for 2 h, the viral supernatants were removed, cells were washed once with PBS, and 2 mL of fresh growth medium was added. Cells were incubated for 5–15 days at 37°C. Viral supernatants (P_1_ stocks) were collected and used for following passages performed as described above; supernatants from each passage (P_2_, P_3_, and P_4_) were collected. FFA was used to quantify the titers of the rescued viruses in the collected virus stocks. mCherry expression was determined based on the signal intensity measurement using a Cellomics ArrayScan VTI microscope. oxGFP expression was detected using an immunofluorescence assay with an anti-GFP antibody (A11122, Invitrogen). For the measurement of NLuc activity, 96-well plate was infected with P_1_ to P_4_ stocks, according to the end-point titration method, and NLuc was measured using the Nano-Glo Luciferase Assay System (Promega) after cell lysis with 1X passive lysis buffer (E1941, Promega), using an EnVision plate reader (Plmer). The percentage of the virus-infected cells expressing reporters was calculated based on the ratio of the number of wells positive for the marker (mCherry, NLuc, or oxGFP) and the number of wells positive for the virus (detected by staining with 4G2 antibody).

### Western blot

Viral supernatant (8 mL) on a 3 mL 20% sucrose cushion was ultracentrifugated for 3 h at 100,000 × g at 4°C using an Optima L80-XP instrument (Beckman Coulter). After ultracentrifugation, the liquid was discarded, the tube was dried with Kimtech paper, and the pellet was resuspended in 80 μL of TNE buffer (10 mM TrisHCl (pH 7.0), 100 mM NaCl, and 1 mM EDTA). The obtained samples were denatured in 1× Laemmli buffer at 95°C for 10 min, and proteins were separated using electrophoresis on a 4–15% mini-protean-TGX-precastgel (Bio-Rad). Proteins were then transferred on nitrocellulose membranes using a semi-dry method using a transblot turbo transfer system (Bio-Rad). The membranes were blocked with 5% skimmed milk powder in Percentage of Tween20 for 30 min and incubated overnight at 4°C with either virus-specific primary capsid antibody (rabbit anti-capsid ZIKV GTX134186 in a 1:10,000 dilution; mouse anti-capsid DENV GTX633632 in a 1:3,000 dilution; and rabbit anti-capsid WNV GTX131947-S in a 1:100 dilution; all antibodies were obtained from GeneTex) or primary anti-pan flavivirus envelope antibody (mouse anti D1-4G2-4-15 [4G2] by NovusBio NBP2-52666 in a 1:1,000 dilution). Membranes were rinsed 3 times for 10 min each time in Percentage of Tween20 prior to incubation with an appropriate secondary antibody (anti-mouse IgG DyLight 800- and anti-rabbit IgG DyLight 800-conjugated antibodies SA5-35521 or SA5-35571 from Invitrogen) for 2 h at room temperature. Membranes were rinsed 3 times for 10 min each time in Percentage of Tween20 before being read on a Li-Cor Odyssey scanner 9120.

### Immunofluorescence analysis

A total of 200,000 Vero cells were grown on 35 mm dishes (Fluorodish, Fisher) and infected at a multiplicity of infection (MOI) of 0.05 with P_0_ (collected at day 5 post-transfection) stocks of all analyzed viruses. Infected cells were incubated for 3 (ZIKV, DENV2, and KUNV) or 4 (DENV4) days, after which they were fixed with 4% PFA (Fisher) for 30 min at 20°C. Fixed cells were rinsed with PBS, permeabilized with 0.1% Triton X-100, diluted in PBS for 5 min, and blocked with 2% BSA. Then, the cells were incubated in PBS supplemented with the pan-flavivirus anti-Env monoclonal antibody 4G2 (NovusBio, dilution 1:1,000) in the presence of 0.05% Saponin (Sigma-Aldrich, France) for 1 h, washed three times with PBS, and incubated with anti-mouse IgG secondary antibodies conjugated with Alexa647 (ab150107, Abcam, 1:1,000 dilution) or Alexa488 (A21202, Invitrogen, 1:10,000 dilution) for 2 h at 4°C in the dark. Nuclei were stained with 10 μg/mL Hoechst 33342 (Invitrogen) for 15 min. The stained cells were washed with PBS and epifluorescence and confocal images were acquired using a Cell-Discoverer 7 microscope (Carl Zeiss SAS, France) at 10× and 25× magnification. Mander’s coefficients were calculated based on the images obtained using Zen Blue software (Zeiss).

### RT-qPCR

The cells were infected with ZIKV and DENV2 and lysed using Luna Cell Ready Lysis reagent (New England Biolabs, United Kingdom). Quantification of viral RNA was performed with cell lysates using virus-specific primers, and their levels were normalized to GAPDH mRNA (Table 1). For these analyses, a Luna Universal One-Step RT-qPCR Kit (New England Biolabs) and a CFX opus real-time 384 system (Bio-Rad) were used. Cycling conditions were as follows: reverse transcription at 55°C for 15 min, followed by an initial polymerase activation at 95°C for 1 min, and then 45 cycles of denaturation at 95°C for 10 s and an annealing/extension at 60°C for 45 s. The calibration of the assay was performed with control plasmids containing sequences encoding ZIKV NS1 and DENV2 capsid proteins (Eurofins Genomics, Germany; GenBank accession numbers KU365778.1 and U87411.1, respectively). Primers were obtained from Eurofins Genomics (Germany).

**Table 1:** Primers used for RT-qPCR for viral RNA quantification.

Gene name	Primer sequences	Product size, bp
ZIKV	F: 5’CCGCTGCCCAACACAAG3’	53
	R: 5’CCACTAACGTTCTTTTGCAGACAT3’	
DENV-2	F: 5’CAGATCTCTGATGAATAACCAACG3’	95
	R: 5’CATTCCAAGTGAGAATCTCTTTGTCA3’	
GAPDH	F: 5’GCTCACTGGCATGGCCTTCCGTG3’	177
	R: 5’TGGAGGAGTGGGTGTCGCTGTTG3’	

### Virus sequencing

Viral RNA (140 μL) from P_0_ stocks collected at 7 days post-transfection were extracted using a QIAamp Viral RNA Extraction Kit (Qiagen). RT-PCRs were performed for each virus using TranscriptII One-Step RT-PCR SuperMix (Transgenbiotech, France) and the primers listed in Supplementary Table S1. Cycling conditions were as follows: reverse transcription at 50°C for 30 min, followed by an initial polymerase activation at 95°C for 5 min, and then 35 cycles of denaturation at 94°C for 30 s, annealing at 60°C for 30 s and an extension at 72°C for 4.5 min. Amplified cDNA fragments were separated by agarose gel electrophoresis and extracted from the gel using a Nucleospin Gel Clean-Up Kit (Macherey-Nagel). DNA sequencing was performed by Plasmidsaurus (Eugene, United States).

### Antiviral assay

NITD008 (Bio-Techne 6045/1) was initially dissolved in 100% dimethyl sulfoxide (DMSO D8418, Sigma) at 10 mM and subsequently diluted in DMEM to the desired concentrations (0.05–15 μM). DMSO (0.5%) was set as the vehicle control. Cells were incubated with increasing concentrations of NITD008 for 2 h prior to infection. For cell fluorescence counting, 9,000 cells per well were cultured in a black opaque 96-well Microplate (PerkinElmer), infected with virus stock at an MOI of 0.01 (ZIKV-mCherry) or MOI 0.05 (DENV2-Ct-mCherry), and prepared for imaging at 72 h post-infection. Briefly, cells were fixed with 4% PFA and stained with 10 μg/mL Hoechst 33342 for 15 min and washed with PBS. Image tiles acquisitions (3 × 2 per well) were performed at a magnification of 2.5 in wide-field on a Cell-Discoverer 7 microscope (Carl Zeiss SAS, France). Image analysis was performed using ZEN Blue software for segmenting of the stained nuclei and mCherry positive cells. The number of nuclei were sorted per well. The sum of fluorescence intensity was weighted against the normalized number of nuclei. For the measurement of NLuc activity, 20,000 cells were plated on a transparent 96-well plate and infected with ZIKV-NLuc at an MOI of 0.05. Luminescence was measured at 1 day post-infection using the Nano-Glo Luciferase Assay System (Promega) on the EnVision plate reader after cell lysis with 1X passive lysis buffer (E1941, Promega). Results were normalized to the total protein amount per well, which was determined using a BCA protein assay kit (Pierce). For wt viruses, 20,000 cells were cultured in a transparent 96-well plate and infected with DENV2-wt or ZIKV-wt at an MOI of 0.01 and 0.05, respectively. Seventy-two hours post-infection, cells were lysed and total viral RNA was extracted and quantified by RT-qPCR, as described above. In all cases, the 50% effective concentration (EC_50_) was calculated by fitting the dose–response curves traced from 4parameter non-linear regression with GraphPad Prism v9.0, based on the following calculations: % Inhibition =100− (drug−cellDMSO/infectedcells−cellDMSO)×100 .

### Transmission electron microscopy

Vero cells were infected at an MOI of 1, incubated for 3 days, fixed with 2.5% glutaraldehyde in PHEM buffer (PIPES 60 mM, HEPES 25 mM, EGTA 10 mM, and MgCl2 2 mM, all provided by Merck-Sigma, Germany), post-fixed in 1% OsO_4_/0.8% K_4_Fe (CN)_6_, and then dehydrated in successive ethanol baths (50/70/90/100%).

Samples were then infiltrated in propylene oxide (MERCK-SIGMA, Germany)/EMbed812 (EMS, USA) mixes, embedded in EMbed812, and polymerized at 60°C. Ultrathin sections (70 nm) were cut using a PowerTome XL ultramicrotome (RMC, Tucson, AZ, United States), stained in 0.2% OTE/lead citrate, and observed on a Tecnai G2 F20 (200 kV FEG) TEM at the platform Plateau de Microscopie Electronique COMET, INM.

### Atomic force microscopy

AFM imaging was performed on a JPK-Bruker Nanowizard IV XP atomic force microscope (JPK BioAFM, Bruker Nano GmbH, Berlin, Germany) operating in BSL-3 (Lyonnais et al., 2021). *Flavivirus* virions were imaged in imaging buffer (10 mM Tris–HCl [pH 7.5] and 100 mM NaCl) shortly after being adsorbed on a freshly cleaved muscovite surface (mica grade v1, Ted Pella) glued on a glass slide. Before virus adsorption, the mica was functionalized with 100 μL of Poly-L-lysine 0.1% (P8920, Sigma) for 10 min, then washed three times in imaging buffer. A three-dimensional printed plastic O-ring was glued around the mica to form a small liquid cell. Viral samples (20 μL) were deposited on the functionalized mica from 15 min to 1 h, and the volume was completed to 200 μL with the imaging buffer. AFM topographic images were obtained using the quantitative imaging (QI) mode using qp-Bio-AC CB2 (Nanosensors), BL-AC40TS (Olympus), or MLCT-Bio (Bruker) cantilevers. Before each set of acquisitions, the sensitivity and spring constant of the cantilever were calibrated (thermal noise method). The applied force was kept at 150–200 pN, 100 nm Z-length, and 20 msec/pixel speed. Using JPK SPM-data processing software, images were flattened with a polynomial/histogram line fit. Low-pass Gaussian and/or median filtering was applied to remove minor noise from the images. The Z-color scale in all images is given as relative after processing. Particle height analysis, based on the height (measured) channel of the QI mode, was performed using the cross section of the analysis software to calculate the maximal central height on each particle.

### Statistical analysis

Statistical analysis was performed using GraphPad Prism 9.3.0 software. The results of marker expression were represented with error bars indicating the standard deviation (SD). One-way ANOVA followed by Tukey’s multiple comparisons test was used for statistical analysis, as indicated in the figures.

## Data availability statement

The datasets presented in this study can be found in online repositories. The names of the repository/repositories and accession number(s) can be found in the article/Supplementary material.

## Ethics statement

Ethical approval was not required for the studies on animals in accordance with the local legislation and institutional requirements because only commercially available established cell lines were used.

## Author contributions

LC, AT, NG, MH, MV, AN, GD, CC-B, AM, and SL performed the experiments. LC and AM constructed the icDNA clones. LC, AT, NG, MH, GD, and CC-B performed virus production, purification, viral titers, sequencing, immunoblots, infection kinetics, FFA, and data analysis. AT and SL performed AFM analysis. MV and SL performed fluorescence microscopy imaging and analysis. AN performed TEM imaging. NG, SL, AM, and DM conceived, directed, and supervised the study. SL, LC, NG, AT, AM, and DM wrote and edited the manuscript. AM and DM raised funding. All authors contributed to the article and approved the submitted version.

## Funding

The BSL3 Bio-AFM was funded by the REDSAIM program at Montpellier University, the BSL3 Cell-Discoverer 7 microscope was funded by Occitanie FEDER. AM and LC were supported by the Estonian Research Council (PRG1154). DM was supported by CNRS, University of Montpellier, Region Occitanie and FEDER EU programme.

## Conflict of interest

The authors declare that the research was conducted in the absence of any commercial or financial relationships that could be construed as a potential conflict of interest.

## Publisher’s note

All claims expressed in this article are solely those of the authors and do not necessarily represent those of their affiliated organizations, or those of the publisher, the editors and the reviewers. Any product that may be evaluated in this article, or claim that may be made by its manufacturer, is not guaranteed or endorsed by the publisher.

## Footnotes

^1^ https://ictv.global/taxonomy
