# Supplementary material for: Validation of flavivirus infectious clones carrying fluorescent markers for antiviral drug screening and replication studies
Source: Front Microbiol. 2023 Sep 15;14:1201640. doi: 10.3389/fmicb.2023.1201640 (PMC10541152; doi:10.3389/fmicb.2023.1201640)

Supplementary Material

Validation of Flavivirus Infectious Clones Carrying Fluorescent Markers for Antiviral Drug Screening and Replication Studies

Liubov Cherkashchenko[1,2]*, Nathalie Gros[1]*, Alice Trausch[1]*, Aymeric Neyret[1], Mathilde Hénaut[1], Gregor Dubois[1], Matthieu Villeneuve[1], Christine Chable-Bessia[1], Sébastien Lyonnais[1], Andres Merits[2#], Delphine Muriaux[1,3#§].

[1] CEMIPAI UAR3725 CNRS, University Montpellier, France

[2] Institute of Technology, University of Tartu, Tartu, Estonia

[3] IRIM UMR9004 CNRS, University of Montpellier, France

[*] *These first authors contributed equally to this article*

[#] *These senior authors were co-principal investigators*

[§] *Corresponding: delphine.muriaux@cnrs.fr; andres.merits@ut.ee*

Supplementary Table S1. Results of the viral genome sequencing (from the PCR products).
Please refer to the file Table 1.xlsx

Supplementary Table S2. Primers used for virus sequencing.

Primer name	Sequence (5' –> 3')	Target region & expected amplicon length *
ZIKV Fw	GCGAAAGCTAGCAACAGTATCAACAGG	5'UTR to NS1-NS2A junction<br>3543 bp
ZIKV Rv	CAAGCACTCCAAGGGAGAAGTGGTCC	
DENV-2 Fw	GAGGGAGCTAAGCTCAACGTAGTTCTAAC	5'UTR to NS2A (5')<br>3474 bp
DENV-2 Rv	GACTCCTAGTGAAAAGTTGTCGACCTGCC	
DENV-4 Fw	CGGAAGCTTGCTTAACACAGTTCTAACAGTTTG	5'UTR to NS2A (5')<br>3494 bp
DENV-4 Rv	CAAACAAGGTCAGGCACAACAGACCC	
KUNV Fw1	GATTTTGAACAATTAACACAGTGCGAGC	5'UTR to Env<br>1999 bp
KUNV Rv1	CACCTCTTGCGAAGGACCTCC	
KUNV Fw2	CAGTACACAGGCACGGATGGAC	Env to NS2A (5')<br>1661 bp
KUNV RV2	GGTTGACGGTAACTAACCTGCCC	

* Product size is provided for the wt sequence, excluding the reporter gene (~ 1 kbp).

Figure S1. Detection of envelope and capsid proteins in purified recombinant flavivirus particles.

A

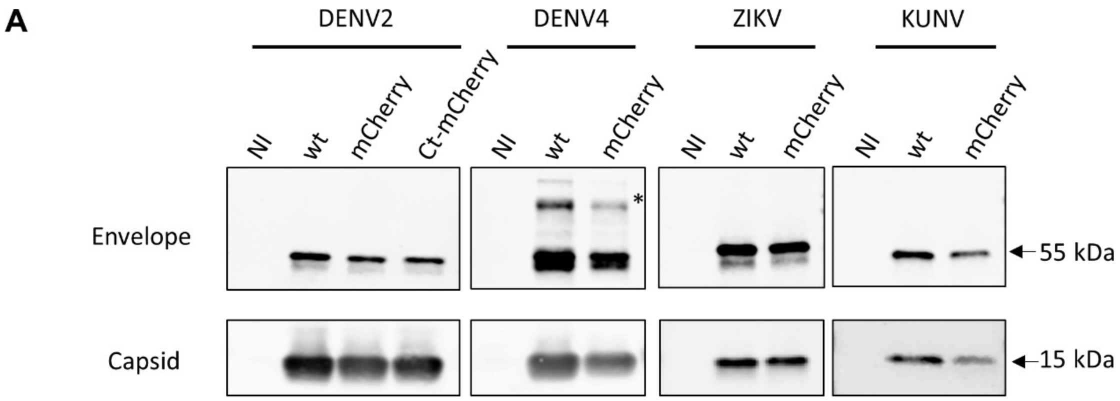

B

Virus	Viral titers (FFU/mL)
DENV2 wt	4,35x10^{7}
DENV2 mCherry	9,38x10^{6}
DENV2-Ct-mCherry	2,97Ex10^{7}
DENV4 wt	2,02x10^{9}
DENV4 mCherry	9,38x10^{7}
ZIKV wt	1,38x10^{9}
ZIKV mCherry	4,35x10^{8}
KUNV wt	1,38x10^{6}
KUNV mCherry	6,39x10^{5}

Supplementary Figure S2: **Fluorescence images of the mCherry signal over time for ZIKV-, KUNV-, DENV2- and DENV4-mCherry expressing constructs** in transfected Vero cells from day 1 up to day 12**.** Scale bars: 100 nm.

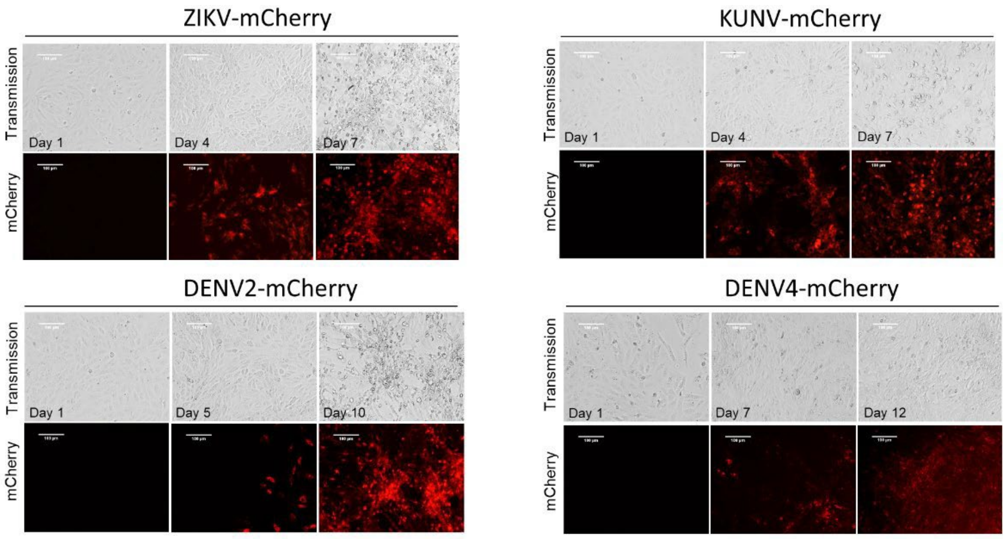

Figure S3. RT-PCR amplification for virus sequencing. Viral RNA were purified from P0 virus stock at 7 days post transfection. 5’-UTR to NS2A fragments (~ 3,5kbp for the WT + ~ 1kbp for the reporter gene for each replicon) were amplified by RT-PCR using the primers listed in Table S1. The amplified DNA were separated on 1% Agarose gel. The amplification products (red squares) were extracted and purified from the agarose gel prior to DNA sequencing. Molecular weights are indicated on the left of each gel. Lane 2: negative control of RT-PCR performed with water. Lane 3: control PCR reaction without the enzyme Reverse Transcriptase (RT).

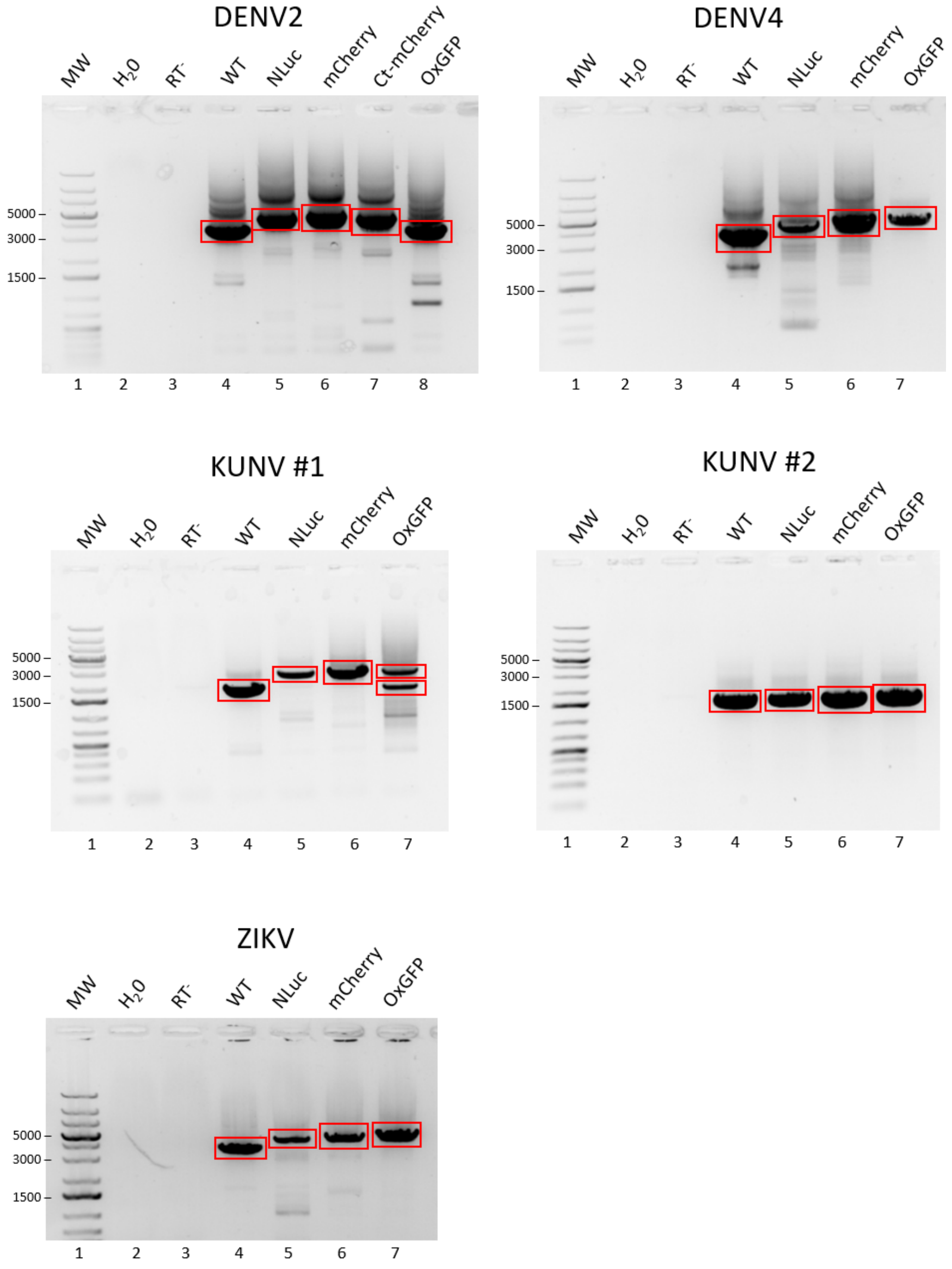

Figure S4. Sequences of Denv2, Denv4 and ZIKV wt and reporter clones collected at 7 days post-transfection.

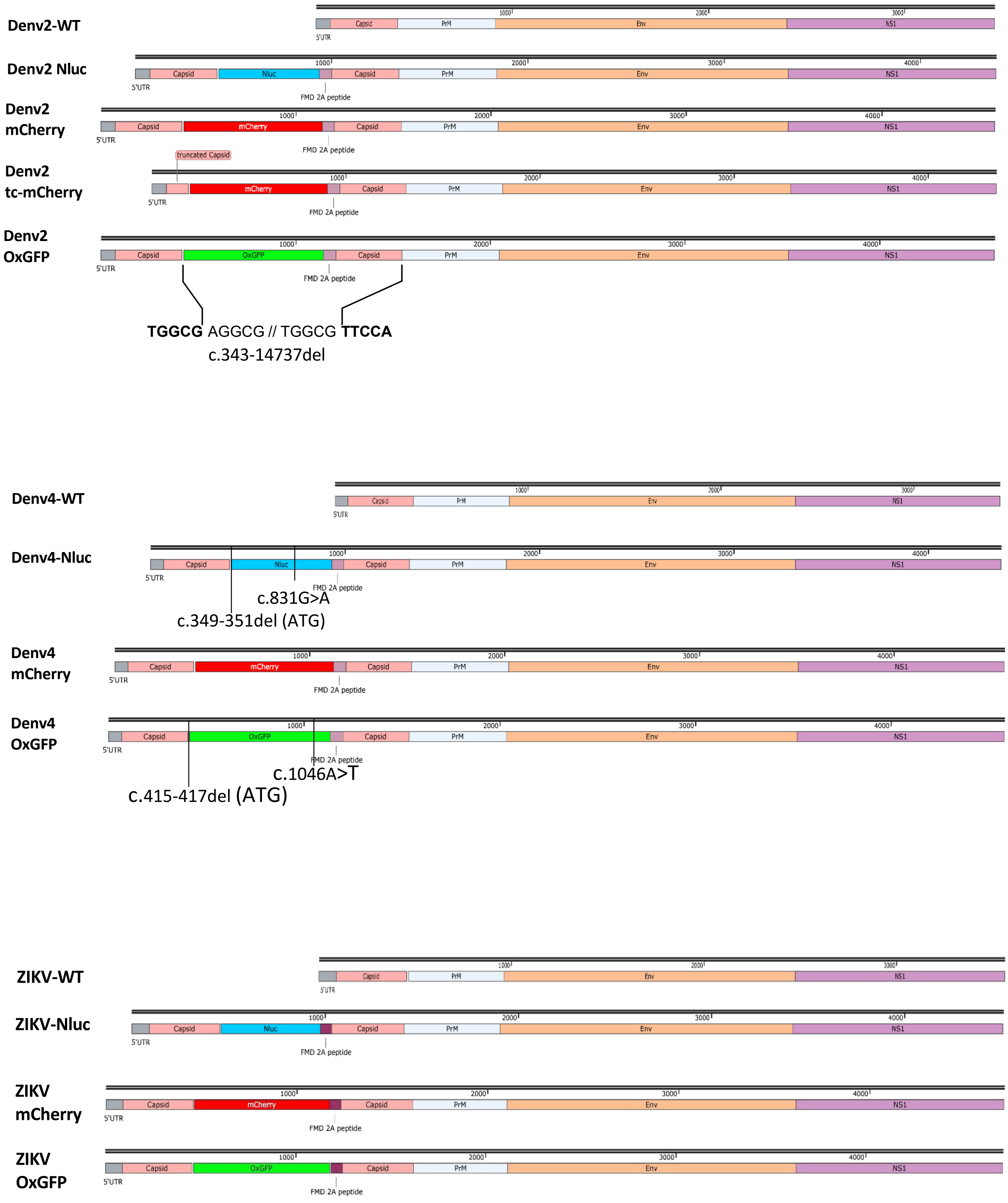

Figure S5. Sequences of KUNV wt and reporter clones collected at 7 days post-transfection.

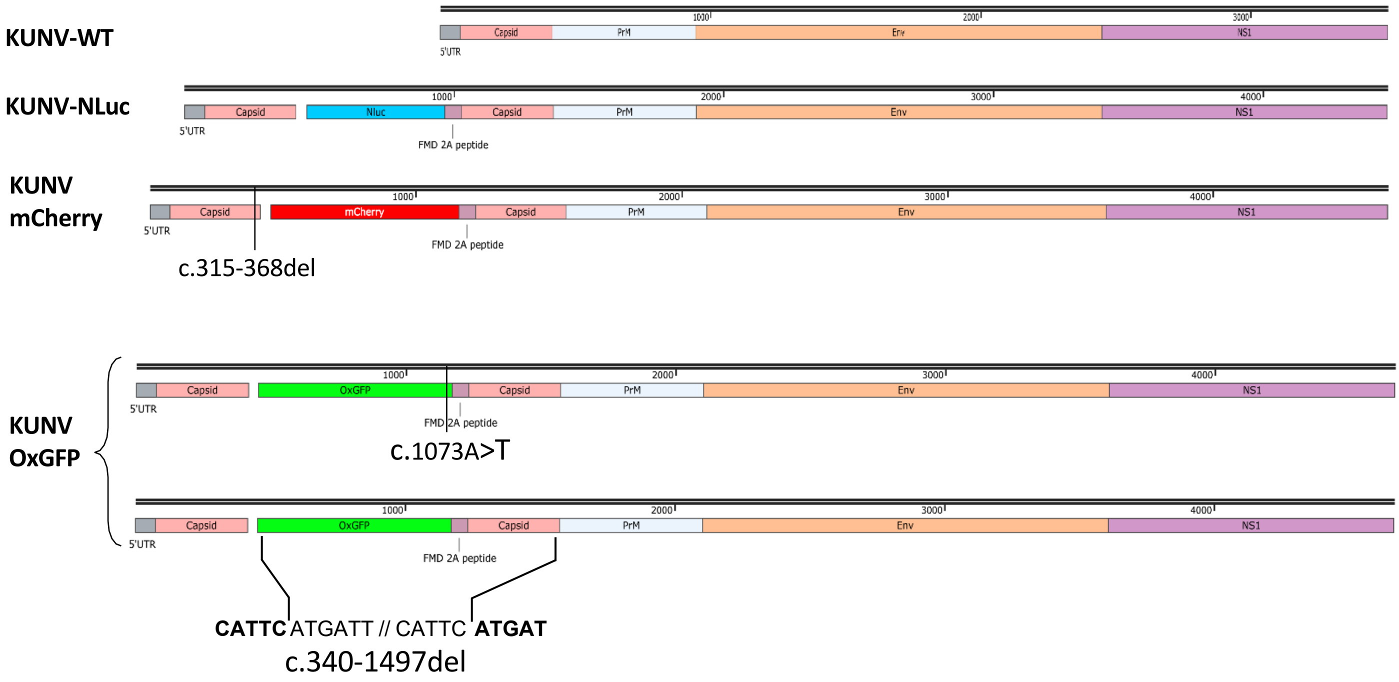

Supplement: Supplementary file 2 [file Data_Sheet_1.pdf]
